# The Internal-to-External Load Ratio: A Tool to Determine the Efficacy of Heat Acclimation/Acclimatization Using Self-Paced Exercise

**DOI:** 10.3389/fspor.2021.830378

**Published:** 2022-01-11

**Authors:** Julian Andro P. Ramos, Carly J. Brade, Kagan J. Ducker, Grant J. Landers, Olivier Girard

**Affiliations:** ^1^Curtin School of Allied Health, Curtin University, Bentley, WA, Australia; ^2^School of Human Sciences (Exercise and Sport Science), University of Western Australia, Crawley, WA, Australia

**Keywords:** endurance, heat acclimation, self-paced, heat acclimatization, ratio

## Introduction

To combat the negative effects of heat on exercise tolerance, daily training for 1–2 weeks for 60–90 min in hot conditions (heat acclimation or acclimatization; HA) is recommended (Periard et al., 2021). Briefly, HA results in physiological (e.g., lower core temperature) and perceptual (e.g., improved thermal comfort) adaptations, which may enhance exercise performance (e.g., increased power output; PO) in the heat (Periard et al., 2021). Heat acclimation protocols typically involve performing continuous or intermittent exercise, either at a fixed intensity (e.g., maintaining a PO corresponding to 60% of maximal aerobic capacity; $\dot{\text{V}}$O_2max_) or using a physiologically controlled approach [i.e., fixed hyperthermia (core temperature ~38.5°C) or heart rate (HR; ~150 bpm); Periard et al., 2021]. Alternatively, self-paced exercise, whereby athletes self-regulate work rate during HA sessions to match a perceptually regulated intensity (e.g., exercise at a given rating of perceived exertion; RPE), is gaining popularity (Gibson et al., 2020; Periard et al., 2021).

## Nature of the Problem

A heat stress test (HST) is typically performed pre- and post-HA to assess the effectiveness of a HA program from changes in physiological, perceptual and performance variables. Interpreting the interactions between these variables and determining what heat-related adaptations have occurred, is easier when work rate is fixed. For example, lower HR post-HA compared to pre-HA when external load is fixed (e.g., cycling at an absolute intensity of 100 W) infers that physiological adaptations have occurred. However, interpreting whether adaptations have been attained during self-paced HA (e.g., 20-km cycling time-trial) is more difficult, as both external and internal loads vary (Periard et al., 2021). For example, it is harder to ascertain whether adaptations have developed when PO (158 vs. 150 W) and HR (169 vs. 160 bpm) hypothetically change in similar proportion post-HA compared to pre-HA.

## Proposal

**Figure 1:**
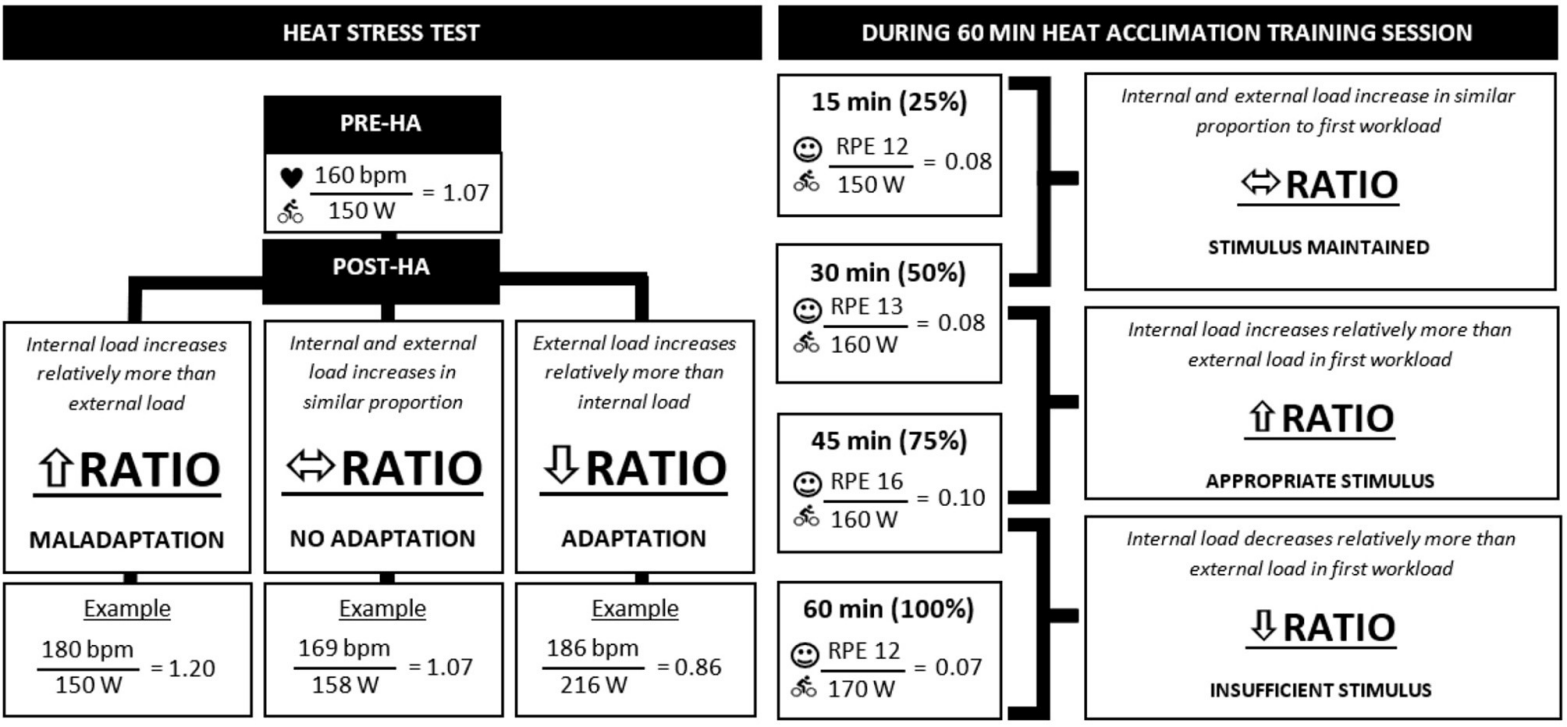
Hypothetical example of applying an internal-to-external load ratio for assessing self-paced heat stress tests and heat acclimation (HA) training sessions. 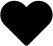 = mean heart rate, 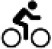 = mean power output, 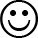 = rating of perceived exertion.

Utilizing an internal-to-external load ratio may be a method of objectively concluding whether a self-paced HA session or protocol is effective (Figure 1) compared to other methods (e.g., observing changes in sweat rate, and core or skin temperature when external load is fixed). Ratios could be applied to physiological or perceptual variables (internal load; HR, thermal comfort or sensation) and performance outcomes (external load; mean PO) obtained during a HST, single HA session, or throughout a HA program.

For example, observing a larger relative change in internal load as opposed to external load (thus a lower internal-to-external ratio) during a post-HA compared to pre-HA HST may be indicative of HA. This is evident in a study by Wingfield et al. (2016) who performed a HST in the form of a 20-km self-paced cycling time-trial in the heat (33.1°C, 60.0% RH) pre- and post-HA training (5 consecutive days cycling for 30 min at alternating intensities every 3 min between 40 and 70% of peak PO in 32°C, RH not reported). Results showed no difference in completion time (40.46 vs. 40.45 min) pre- and post-HA HST, which may indicate that no adaptation to the heat had been attained. However, the internal-to-external load ratio on mean PO (154 vs. 157 W) and HR (161 vs. 153 bpm) pre- and post-HA HST displays lower values post-HA compared to pre-HA (0.98 vs. 1.05). This is due to a lower HR despite a higher sustained PO post-HA, suggesting that HA has occurred as a lower internal-to-external ratio compared to pre-HA HST is observed.

Alternatively, internal-to-external load ratios could be utilized to determine the efficacy of a single session of self-paced HA training. For example, if internal-to-external load ratio for a hypothetical RPE (12 vs. 13) and mean PO (150 vs. 160 W) at 25 and 50% of total exercise time completed show similar ratios (0.08), this indicates that the heat stimulus has been maintained throughout the session. Alternatively, reductions or failure to maintain ratios throughout a single self-paced HA session could indicate an ineffective session. This may be due to the internal load decreasing relatively more than the external load, which indicates that the athlete is not receiving the stimulus required to induce heat adaptations.

Finally, whole-session internal-to-external load ratios may be utilized to track whether athletes are receiving the appropriate stimulus for heat adaptation throughout a HA program. For instance, if a hypothetical whole-session rating of thermal sensation (15; 0–20 scale; Gaoua et al., 2012) and mean PO (180 W) were obtained for the first session of a HA program (ratio = 0.08), subsequent sessions in a simple stepwise progression will need to obtain a ratio of ≥0.08 to ensure athletes are receiving the appropriate progressive overload stimulus.

## Conclusion

Utilization of internal-to-external load ratios could assist with objectively concluding that a self-paced HA session or protocol is effective at inducing required heat adaptations. This could lead to a novel addition in identifying the effectiveness of HA protocols.

## Author Contributions

All authors contributed to all elements of the research and read and approved the manuscript.

## Funding

This paper was completed under the support of the Australian Government Research Training Program Scholarship.

## Conflict of Interest

The authors declare that the research was conducted in the absence of any commercial or financial relationships that could be construed as a potential conflict of interest.

## Publisher's Note

All claims expressed in this article are solely those of the authors and do not necessarily represent those of their affiliated organizations, or those of the publisher, the editors and the reviewers. Any product that may be evaluated in this article, or claim that may be made by its manufacturer, is not guaranteed or endorsed by the publisher.
